# The CXC Chemokine Receptors in Four-Eyed Sleeper (*Bostrychus sinensis*) and Their Involvement in Responding to Skin Injury

**DOI:** 10.3390/ijms221810022

**Published:** 2021-09-16

**Authors:** Mengdan Dong, Hong Zhang, Chengyu Mo, Wenjing Li, Wanwan Zhang, Kuntong Jia, Wei Liu, Meisheng Yi

**Affiliations:** 1 School of Marine Sciences, Sun Yat-sen University, Zhuhai 519082, China; dongmd5@mail2.sysu.edu.cn (M.D.); zhangh395@mail2.sysu.edu.cn (H.Z.); mochy5@mail2.sysu.edu.cn (C.M.); liwj77@mail2.sysu.edu.cn (W.L.); zhangww56@mail2.sysu.edu.cn (W.Z.); jiakt3@mail.sysu.edu.cn (K.J.); 2 Southern Marine Science and Engineering Guangdong Laboratory, Zhuhai 519082, China; 3 Guangdong Provincial Key Laboratory of Marine Resources and Coastal Engineering, Guangzhou 510275, China

**Keywords:** four-eyed sleeper, CXC chemokine receptors, CXCR6, skin injury

## Abstract

CXC Chemokine signaling plays an important role in wound healing. The four-eyed sleeper (*Bostrychus sinensis*) is a commercially important marine fish, which is prone to suffer skin ulceration at high temperature seasons, leading to mass mortality of fish in aquaculture farms. The genetic background related to skin ulceration and wound healing has remained unknown in this fish. Herein, we identified 10 differentially expressed *Bostrychus sinensis* CXC chemokine receptors (*BsCXCRs*) in skin ulcerated fish by de novo transcriptome sequencing. The transcripts of these *BsCXCRs* were classified in seven types, including *BsCXCR1a/1b*, *BsCXCR2*, *BsCXCR3a1/3a2*, *BsCXCR4a/4b*, and *BsCXCR5-7*, and *BsCXCR6* was the first *CXCR6* homologue experimentally identified in teleost fish. These BsCXCRs were further characterized in gene and protein structures, as well as phylogenetics, and the results revealed that BsCXCRs have expanded to divergent homologues. Our results showed that, in healthy fish, the *BsCXCR* transcripts was mainly distributed in the muscle and immune related organs, and that BsCXCR1a/1b proteins located in the cytomembrane, BsCXCR4a/4b/5/6 in the cytomembrane and perinuclear region, and BsCXCR3a1/3a2/7 in the cytomembrane, perinuclear region, and nuclear membrane, respectively. In skin injured fish, the transcripts of all *BsCXCRs* were transiently increased within one hour after injury, suggesting the involvement of *BsCXCRs* into the early inflammatory response to skin injury in the four-eyed sleeper. These results are valuable for understanding the evolutionary events of fish *CXCR* genes and provide insights into the roles of CXCR family in fish skin injury.

## 1. Introduction

Commercial aquaculture is one of the most important sources of food, especially of high-quality protein from farmed fish. However, fish culture has become increasingly vulnerable to various aquatic environmental stressors, including pathogens and pollution, in the intensive cultivation system. The skin is the first barrier against these factors [1,2], and skin damage of fish may result in pathogen infection, which eventually causes fish death. Most cutaneous injuries accompanied by vascular damages trigger an acute inflammatory response [3] and are followed by wound healing, which is a dynamic, interactive process consisting of three phases, inflammation, tissue formation, and tissue remodeling, with the involvement of various soluble mediators, extracellular matrix, resident cells, and leukocytes [4,5]. Inflammation is the first critical step of wound healing, which happens in the first several hours of injury for preventing ongoing blood and fluid losses, as well as pathogen infection. Some reports reveal that the chemokine-receptor system is involved in inflammation, angiogenesis, and leukocyte recruitment, as well as contributed to the epithelialization during wound repair [6,7]. Chemokine receptors, according to the ligands they bind to, are classified into 4 subfamilies, CCR, CXCR, CX3CR, and XCR [8]. Upon binding to their cognate ligands, receptors undergo conformational change that transmits signals to initiate a hierarchical signal transduction [9,10,11]. CXC chemokine signaling plays important roles in vascular, larval fin regeneration and skin repair by recruiting immune cells including neutrophils and macrophages, in wound-induced inflammatory response. The CXCL12-CXCR4 chemokine signaling plays an important role in wound-induced inflammation by increasing the density of macrophages to clear debris from sites of tissue damage, or by recruiting endothelial progenitor cells to promote epithelialization [7,12,13]. In zebrafish, mutation of either *CXCL12* or *CXCR4b* decreases the number of neutrophils in kidney and exacerbates the recruitment of neutrophils to wounds, demonstrating that CXCL12-CXCR4b signaling is required for granulopoiesis and dynamic control of neutrophil migration [14]. Medaka *CXCR3A* is a hallmark of mononuclear phagocytic cells, and the expression of *CXCR1*, *CXCR3A*, *CXCR4B*, and *CXCR5* transcripts is rapidly accumulated near the wound in the tail fin [6]. These results indicate that CXCR family provide significant contributions in wound-induced inflammation and are required for wound repair. However, the expression patterns and putative functions of CXCR family remain poorly understood in most fish species, especially in commercial farmed fish.

The four-eyed sleeper (*Bostrychus sinensis*) is a kind of burrowing marine fish inhabiting along the inshore of the Indo-Pacific districts from India to Australia, China, and Japan [15,16]. During breeding season, the female enters the borrow to lay eggs and leaves the burrow, and then the male remains to guard the eggs [16,17]. This fish has been commercially cultured in China and Southeast Asian countries due to its rapid growth, high viability to be transported, and its potential as nourishing food in speeding wound healing after surgery [17]. However, it was found that the four-eyed sleeper was prone to suffering skin injury during high temperature season, which eventually led to fish death. Nevertheless, the genetic background of this fish for responding to skin injury, and a better understanding of these responses, would be helpful in prevention and control of this disease. In this study, we identified 10 *Bostrychus sinensis* CXC chemokine receptors (*BsCXCRs*) that were differentially expressed in skin ulcerated fish by transcriptome analysis, and we globally investigated their phylogenetic evolution, gene and protein characteristics, and dynamic expression in response to skin injury. Moreover, we observed a transient and rapid increase of BsCXCR family in early inflammatory response to mechanical skin injury. These data provide essential data for investigating the role of CXCR family in skin ulceration and will be helpful for developing drugs or fish manure to control this disease.

## 2. Results

### 2.1. Transcriptome Analysis of the Healthy and Skin Ulcerated Fish

**Figure 1:**
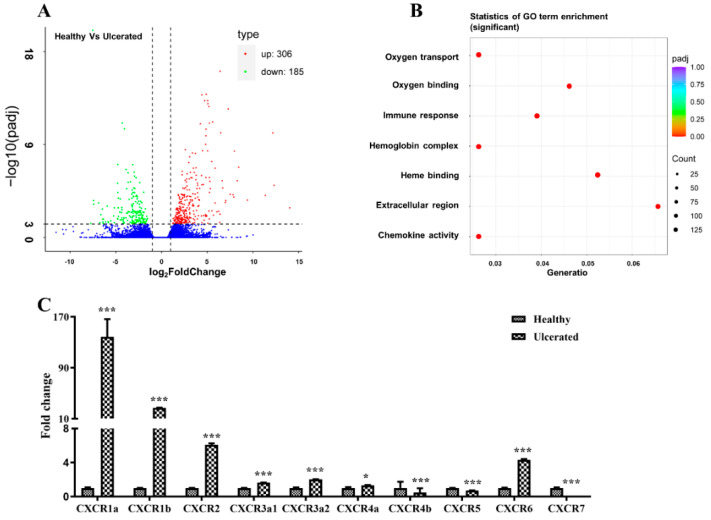
DEGs in healthy and ulcerated four-eyed sleeper and qPCR validation. (**A**) Volcano plot of the DEGs. (**B**) The Go term enrichment of DEGs. (**C**) qPCR analysis of CXCR members in the spleen of healthy and ulcerated. The ordinate represents the relative fold change, the expression level of CXCR in healthy fish was normalized as one (* *p* < 0.05; *** *p* < 0.001).

Skin ulceration is one of the severest skin injuries, which remains poorly understood in most fish species. To identify the genes associated with skin ulceration, we performed de novo transcriptome sequencing in healthy and skin ulcerated four-eyed sleeper. The transcriptome assembled 110,918 unigenes, including 491 DEGs, which were significantly enriched in GO terms of oxygen transport, oxygen binding, immune response, hemoglobin complex, heme binding, extracellular region, and chemokine activity (Figure 1A,B), and these GO terms are closely associated with skin wound repair [2]. Of these DEGs, we identified 10 CXCR transcripts, and qPCR examination indicated the significant up-regulation of *BsCXCR1a*/*1b*/*2*/*3a1*/*3a2*/*4a*/*6* and down-regulation of *BsCXCR4b*/*5*/*7* in skin ulcerated fish compared to healthy fish.

### 2.2. Sequence Analysis of CXCRs

**Figure 2:**
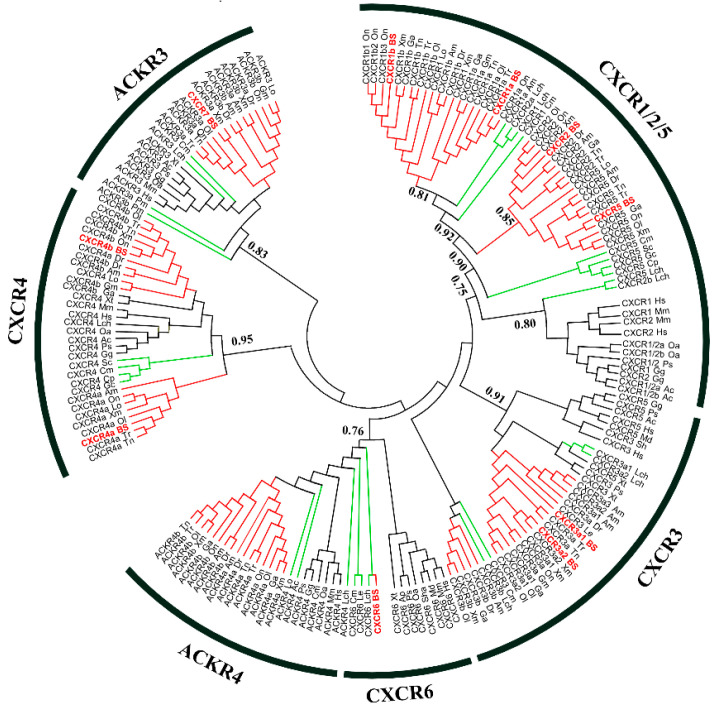
Phylogenetic analysis of vertebrate CXC chemokine receptors. Posterior probability values were included for the major nodes from supporting maximum likelihood analysis (values below 0.5 were not shown). The teleost fish are indicated in red; lower fish species including *Leucoraja_erinacea* (jawless fish), shark (cartilaginous fish), and Coelacanth (*Latimeria_chalumnae*), are shown in green; and non-fish vertebrates are shown in black.

Several studies have reported that *CXCR6* is absent in teleost fish [18,19,20]. However, of these 10 four-eyed sleeper *CXCR* transcripts, one was predicted as *CXCR6*. To obtain an explicit annotation, a phylogenetic tree was constructed based on previously identified amino acid sequence of vertebrate CXCR homologs. Overall, the phylogenetic analysis supported the identification because all BsCXCRs, except for BsCXCR6, were clustered together with corresponding teleost fish CXCR homologues. The BsCXCR6 was clustered together with cartilaginous fish CXCR6 homologues (Figure 2). Therefore, *BsCXCR6* is the first experimentally identified *CXCR6* gene in teleost fish. Additionally, we found that many teleost fish, including four-eyed sleeper, had at least two copies of CXCR1/3/4 homologues. 

**Figure 3:**
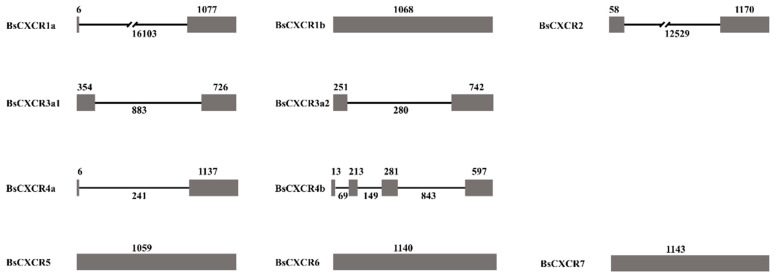
The exon-intron arrangements of *BsCXCR* genes. The grey boxes and black line represent the exons and introns, respectively. The number indicate their length.

Subsequently, we characterized gene and protein structures of BsCXCRs family. The exon-intron arrangements are highly variable in *BsCXCRs* genes, even in duplicated paralogs (Figure 3).

**Figure 4:**
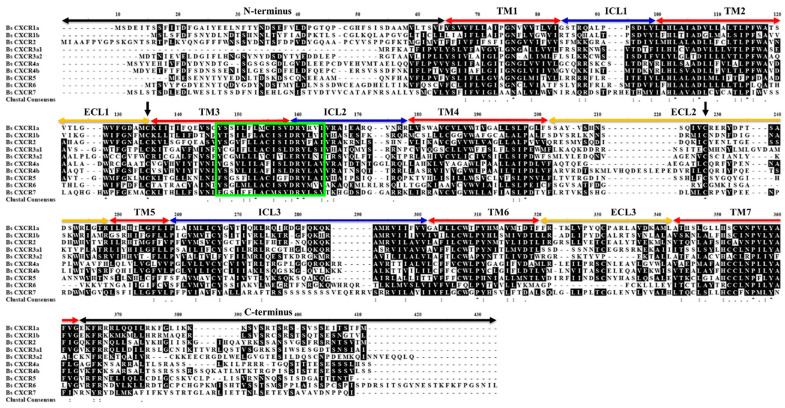
Multiple amino acid sequence alignment of BsCXCR1-7 proteins. TM, transmembrane domain; ECL, extracellular loops; ICL, intracellular loops. Black arrows indicate the conserved cysteine residues. The signature region is shown in the box.

*BsCXCR1a* has two exons and one intron, whereas *BsCXCR1b* is intronless; *BsCXCR4a* has two exons and one intron, whereas *BsCXCR4b* has four exons and three introns. *BsCXCR2*/*3a1*/*3a2* have two exons and one intron, and *BsCXCR5*/6/*7* are all intronless. Multiple alignments indicated that all BsCXCR proteins have seven transmembrane domains (TM), three extracellular loops (ECL), three intracellular loops (ICL), and a DRY signature motif (Figure 4).

In addition, the number of N-glycosylation sites was also variable in these BsCXCRs (Table 1).

**Figure 5:**
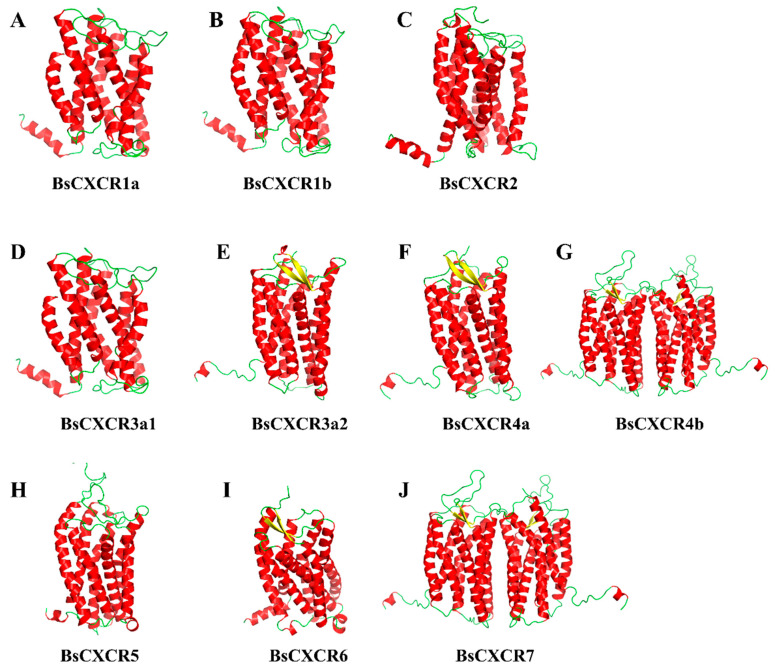
The predicted 3D structures of BsCXCR proteins. (**A**) BsCXCR1a, (**B**) BsCXCR1b, (**C**) BsCXCR2, (**D**) BsCXCR3a1, (**E**) BsCXCR3a2, (**F**) BsCXCR4a, (**G**) BsCXCR4b, (**H**) BsCXCR5, (**I**) BsCXCR6 and (**J**) BsCXCR7. α-helices and β-sheets are labeled in red and yellow, respectively.

By using PyMol, the 3D-structures of BsCXCR proteins were predicted to reveal the structural similarities and differences. The prediction showed that all BsCXCRs had seven *α*-helices representing the seven TM domains and that BsCXCR3a2/4a/4b/6/7 had two additional *β*-sheets (Figure 5E,F,I,J). BsCXCR1a/1b/2/3a1/3a2/4a/5/6 proteins were existed as monomer, whereas BsCXCR4b/7 proteins were homodimers, and BsCXCR1a/1b/2/3a1 and BsCXCR4b/7 had relatively highly identical structures, respectively (Figure 5G,J).

### 2.3. Cellular Localizations and Tissue Distributions of BsCXCRs

**Figure 6:**
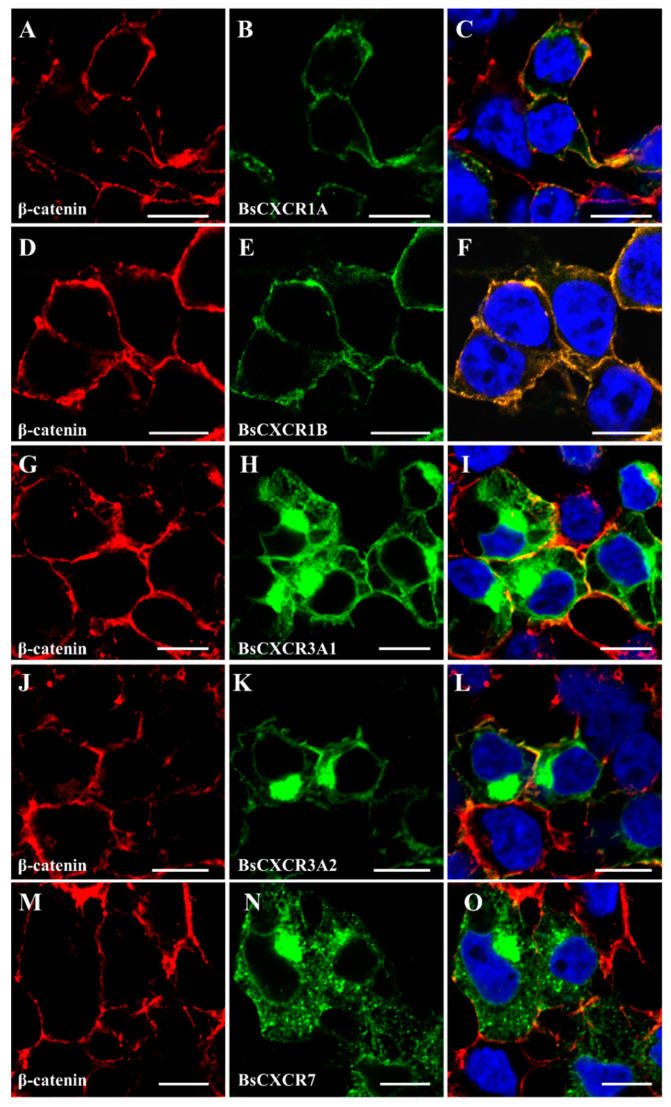
Subcellular localization of BsCXCR1a/1b, BsCXCR3a1/3a2 and BsCXCR7. The indicated recombinant plasmids were transfected in HEK293T cells. The cytomembrane was labeled by anti-*β*-catenin antibody using immunofluorescence. (**A**,**D**,**G**,**J**,**M**) β-catenin, (**B**) *pEGFP-N3-BsCXCR1a*, (**E**) *pEGFP-N3-BsCXCR1b*, (**H**) *pEGFP-N3-BsCXCR3a1*, (**K**) *pEGFP-N3-BsCXCR3a2*, (**N**) *pEGFP-N3-BsCXCR7*, and (**C**,**F**,**I**,**L**,**O**) merged images. Scale bar, 10 μm.

**Figure 7:**
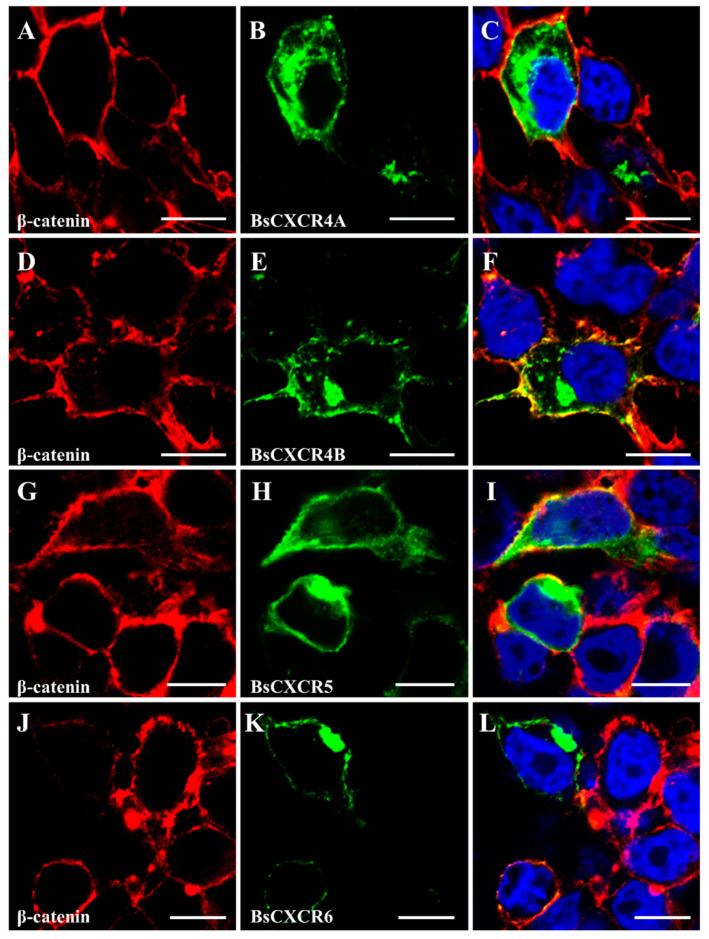
Subcellular localization of BsCXCR4-6. The indicated recombinant plasmids were transfected in HEK293T cells. The cytomembrane was labeled by anti-β-catenin antibody using immunofluorescence. (**A**,**D**,**G**,**J**) β-catenin, (**B**) *pEGFP-N3-BsCXCR4a*, (**E**) *pEGFP-N3-BsCXCR4b*, (**H**) *pEGFP-N3-BsCXCR5*, (**K**) *pEGFP-N3-BsCXCR6*, and (**C**,**F**,**I**,**L**) merged images. Scale bar = 10 μm.

To characterize the subcellular localization of BsCXCR proteins, GFP fusion plasmids of *pEGFP-N3-BsCXCR* were transiently transfected in HEK293T cells, and the cytomembrane was labeled by immunofluorescence staining of β-catenin. BsCXCRs’ localizations were classified in three types. BsCXCR1a/1b were completely co-localized with β-catenin, demonstrating that they were specifically localized at the cytomembrane (Figure 6A–F); BsCXCR3a1/3a2/7 were expressed at the cytomembrane, perinuclear region, and nuclear membrane (Figure 6G–O); and BsCXCR4a/4b/5/6 were expressed in the cytomembrane and perinuclear region (Figure 7). 

**Figure 8:**
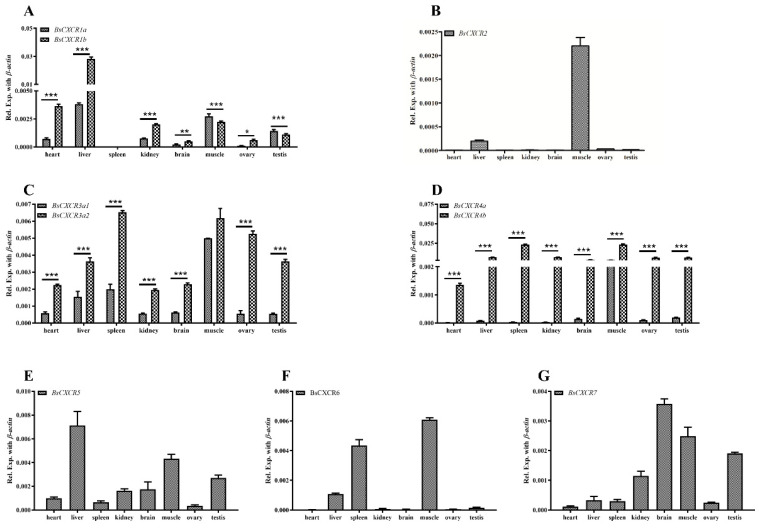
Distribution of *BsCXCR* transcripts in adult four-eyed sleeper tissues. Quantitative RT-PCR (qRT-PCR) analysis of *BsCXCR* transcripts in eight adult tissues. (**A**) *BsCXCR1a/1b*, (**B**) *BsCXCR2*, (**C**) *BsCXCR3a1/3a2*, (**D**) *BsCXCR4a/4b*, (**E**) *BsCXCR5*, (**F**) *BsCXCR6*, (**G**) *BsCXCR7.* Each bar represents mean ± SD (n = 3). The asterisks indicate the significant differences between two groups (* *p* < 0.05; ** *p* < 0.01; *** *p* < 0.001).

The distributions of *BsCXCR* transcripts in adult four-eyed sleeper tissues were examined by qPCR. The results indicated that *BsCXCR* transcripts were abundant in the spleen, liver, muscle, and brain (Figure 8). Even though the duplicated paralogs of *BsCXCR1/3/4* had similar tissue distributions, the expression exhibited significantly biased in adult tissues. The expression levels of *BsCXCR1b* and *BsCXCR3a2* were significantly higher (2–7-folds) compared to *BsCXCR1a* and *BsCXCR3a1*, respectively (Figure 8A,C); and the expression level of *BsCXCR4b* was remarkably higher (33–685-folds) compared to *BsCXCR4a* (Figure 8D). 

### 2.4. Dynamic Expression of BsCXCR Transcripts in Response to Skin Injury

**Figure 9:**
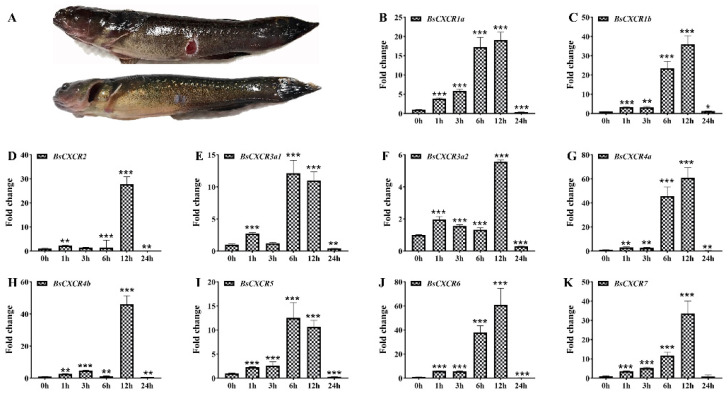
Dynamic expression patterns of *BsCXCR* during skin injury. (**A**) Representative image of four-eyed sleeper at 1 h (up) and 15 d (low) after injury, respectively. (**B**–**K**) The relative fold change of *BsCXCR* transcripts in the wounding sites of the skin at different times after injury (* *p* < 0.05; ** *p* < 0.01; *** *p* < 0.001).

Because *CXCR* transcripts were enriched in immune related organs and muscle, and they were differentially expressed in healthy and skin ulcerated fish (Figure 1 and Figure 8), we speculated that *CXCR* participated in skin injury in four-eyed sleeper. To investigate the involvement of *BsCXCR* in skin injury, we introduced mechanical skin injury and analyzed their expression before or shortly after skin injury (Figure 9A). The transcript levels of all *BsCXCRs* were significantly increased as early as 1 h after injury, reaching a maximal increase within 6–12 h, and then decreased at 24 h (Figure 9B–K). Of these *BsCXCR* transcripts, *BsCXCR4a* and *BsCXCR6* had a maximum of 60-folds increase, suggesting that *BsCXCR4a* and *BsCXCR6* might play an important role in skin injury.

## 3. Discussion

Numerous studies have demonstrated that mammal CXC chemokines regulate the proliferation and differentiation of normal keratinocytes in inflammatory response to skin injury [3]. Although CXCR homologues have been cloned and characterized in several fish species [10,19,21,22,23,24], it remains a lack of a global characterization of CXCR family and their involvement in skin injury in gobies. In this study, we identified 10 differentially expressed *CXCR* transcripts from skin ulcerated four-eyed sleeper (Figure 1), which were classified in seven types. *CXCR* is one of the evolutionary innovations in vertebrate [8]. Zou et al. have classified the fish *CXCRs* in three distinct groups: Agnatha (jawless fish), Chondrichthyes (cartilaginous fish), and Osteichthyes (teleost fish) [18]. The jawless fish sea lamprey has four *CXCR* genes (*CXCR4*, *CXCR7a/7b*, and one *CXCR* related to *CXCR1*/*2*) [20,25]. After second round genome duplication, cartilaginous fish, such as elephant shark, have developed more *CXCR* genes (*CXCR1*/*2*/*4*/*5*/*6*) to adapt the emergence of RAG-mediated adaptive immune system [26]. Due to the teleost specific third round genome duplication, many teleost fish species have duplicated more than two copies of *CXCR* genes, such as *CXCR1*, *CXCR3*, and *CXCR4* [27]. Therefore, cartilaginous fish have all the known CXCR homologues (*CXCR1*–*6*) found in mammals, and teleost fish possess two or more copies of *CXCR1/3/4* genes. Notably, *CXCR6* is absent in teleost fish, to date. In this study, we identified a four-eyed sleeper *CXCR6* gene, which is clustered with cartilaginous fish *CXCR6* and possesses the traditional domains of CXCR proteins (Figure 2, Figure 4 and Figure 5). To the best of our knowledge, *BsCXCR6* is the first experimentally-identified *CXCR6* gene in teleost fish.

Similar to other teleost fish, the exon-intron arrangement is variable in *CXCR* genes, even in duplicated paralogs (Figure 3). Asian swamp eel, rock bream, and four-eyed sleeper *CXCR1a* genes are comprised of two exons and one intron [10,28], whereas four-eyed sleeper and grouper *CXCR1b* genes are intronless [19]. The different exon-intron arrangements of *CXCR4a* and *CXCR4b* are conserved in teleost fish, including four-eyed sleeper [9,22,23,29]. The genomic divergences of duplicated genes might be due to the expansion of chemokine receptors during teleost specific genome duplication.

Because chemokines have high affinity to chemokine receptors, several chemokines can bind to one or two chemokine receptors. For example, the interactions of CXCL8-CXCR1, CXCL8-CXCR2, CXCL12-CXCR4, and CXCL12-CXCR7 have been demonstrated to be critical for directing immune response, wound repair and cell migration in mammals [9,20,30]. However, the evidence of chemokine and receptor binding in fish remains limited. In this study, we predicted BsCXCRs’ structures and found that BsCXCR1a/1b/2/3a1 and BsCXCR4b/7, respectively, have similar structures, suggesting that they might bind to the same chemokine, such as CXCL8 and CXCL12. In mammals, CXCR6 is the sole chemokine receptor of CXCL16 ligand; however, we failed to identify *CXCL16* in either genome or transcriptome data in four-eyed sleeper, and BsCXCR6’s structure was different to other BsCXCRs. Therefore, to which chemokine ligand BsCXCR6 binds needs to be further identified. The localization of the chemokine receptors is intrinsically linked to the role in the structural and functional organization of the cell. However, the subcellular localization of most CXCR members is largely unknown in fish. In this study, we analyzed subcellular localization of BsCXCRs in four-eyed sleeper, and BsCXCRs’ localizations were defined in three groups in the absence of binding to chemokines. We found that BsCXCR1a/1b/3 and BsCXCR4b/7 proteins were predicted to share similar 3D-structures (Figure 5), but their subcellular localizations are different (Figure 6 and Figure 7), suggesting that different CXCRs might cooperatively respond to chemokine signaling. 

Paralogs usually show a dominant, biased, or silencing expression pattern [31,32,33]. Biased expression of *CXCR* paralogs has been reported in several fish species. The expression levels of Asian swamp eel *CXCR1b* and *CXCR3a* are significantly higher than their duplicated paralogs of *CXCR1a* and *CXCR3b*, respectively [10]. The biased expression of *CXCR4a*/*4b* has been observed in zebrafish embryos [11], Asian swamp eel [10], and grouper tissues [9]. Herein, we also observed that the expression levels of *BsCXCR1b*/*3a2*/*4b* were significantly higher than their corresponding paralogs of *BsCXCR1a*/*3a1*/*4a*, respectively (Figure 8), suggesting potentially functional divergence in duplicated *BsCXCR* paralogs.

CXCRs are required for early inflammation during wound repair in mammals. CXCR1 and CXCR2 play a critical role in the recruitment of leukocytes at the site of inflammation shortly after injury [2,34,35]. Loss of *CXCR2* delays the epithelialization and neovascularization during skin repair [36]. The CXCL12-CXCR4 signaling is critical for healing the fracture, as well as neural and cutaneous injury [7,37,38]. Medaka *CXCR3a* is the hallmark of mononuclear phagocytic cells, and the CXCR3a expressing cells are accumulated in the wounds after fin injury [6]. Our results showed that the expression of all *BsCXCRs* was significantly increased in 1 h after skin injury, and it peaked within 6–12 h (Figure 9). The acute and transient increase demonstrated that *BsCXCR* should participate in early inflammation in response to skin injury. However, the exact role of BsCXCR in skin injury would be valuable for further investigation.

In summary, we identified 10 *CXCR* genes especially of the first experimentally—identified teleost *CXCR6* gene *BsCXCR6*, and globally analyzed their similarities and divergences in genome and protein structure, tissue distribution, and subcellular localization. To the best of our knowledge, it is the first study globally characterizing the structure, expression, and localization of *CXCR* family in gobies and intertidal fishes. Furthermore, we observed the differential expression of *BsCXCR* transcripts in healthy and skin ulcerated fish and the significant and transient increase of *BsCXCRs* shortly after skin injury, demonstrating the involvement of *CXCRs* in inflammatory response to skin injury. However, which kind of immune cells *BsCXCRs* express in, and their potential roles, as well as by which mechanism CXCRs use to, in skin injury remain limited. These will be further investigated in our future studies. Ultimately, the present study provides essential data for study of the role and mechanism of CXCR in skin injury in four-eyed sleeper.

## 4. Materials and Methods

### 4.1. Ethical Procedures

All experimental procedures with four-eyed sleeper were approved by the Ethics Committee of Sun Yat-sen University (2020A1515010358), and the methods were in accordance with the approved guidelines. 

### 4.2. Fish, Cells and Sample Collection

Four-eyed sleeper was purchased from the Longhai Ruiquan Aquaculture Cooperation Group (Fujian, China). Thirty 4-month-old individuals with average body length of 9.5 ± 0.2 cm and body weight of 14.1 ± 3.23 g were maintained in circle tanks (150 cm in height and 120 cm in diameter) with 500 l of filtrated seawater (salinity = 12 ppt, oxygen level = 3 mmg/L) at 28 °C under a 14 h/10 h light-dark photoperiod. The aquaculture water was replaced every two days. Fish were fed with fish manure twice a day at 8:30 and 18:00, respectively. 

For RNA sequencing, the spleen tissues were collected independently from three healthy and three skin ulcerated fish, respectively. For tissue distribution analysis, eight tissues (heart, liver, spleen, kidney, brain, muscle, ovary, and testis) were collected for three healthy individuals.

HEK 293T cells were cultured in Dulbecco’s modified Eagle’s medium (DMEM) with 10% FBS (Invitrogen, Carlsbad, CA, USA) under a humidified atmosphere of air containing 5% CO_2_ at 37 °C as previously described [39].

### 4.3. Total RNA Isolation and Illumine Sequencing

Total RNA was isolated by using Trizol reagent (Invitrogen, Carlsbad, CA, USA) according to the manufacturer’s instruction. High-quality RNA (28S/18S ≥ 1.8, OD260/280 ≥ 1.8, and RIN value ≥ 7) was subjected to construct cDNA library for de novo transcriptome sequencing. The de novo transcriptome sequencing of the spleens of three healthy and three skin injured fish was performed on the MGIseq2000 platform (GrandOmics, Wuhan, China). 

### 4.4. TranscriptomeSsequencing and Analysis

After sequencing, raw reads were filtered to get the high-quality clean reads by the fastp (https://github.com/OpenGene/fastp, version 0.22.0, 17 August 2021). The remaining clean reads were assembled by Trinity (https://github.com/trinityrnaseq, version 2.11.0, 17 August 2021) [40]. The assembled sequences were annotated to known public databases, including the NR, Swiss-Prot, Gene Ontology (GO; http://www.geneontology.org, 1 January 2021), euKaryotic Ortholog Groups (KOG), and Kyoto Encyclopedia of Genes and Genomes (KEGG, http://www.genome.ad.jp/kegg/, Release 97.0, 1 January 2021). Differentially expressed genes (DEGs) between healthy and injured spleens were analyzed using the DESeq2 by its adjusted *p*-value ≤ 0.05 and the fold change ≥ 2.0 [41]. GO and KEGG functional enrichment analyses of DEGs were performed by R package cluster profiler [42]. 

### 4.5. Bioinformatic Analysis of BsCXCR Family 

Multiple alignments were analyzed by the Bioedit (version 7.0). The domains of BsCXCRs’ amino acids were predicted by Simple Modular Architecture Research Tool (http://smart.embl-heidelberg.de/, version 9.0, 17 August 2021). The structures of BsCXCR proteins were predicted by PyMOL (version 2.0.6). The N-glycosylation site, functional cysteine and signature motif were predicted by ExPASy (https://prosite.expasy.org/, version 20.0, 17 August 2021). The phylogenetic tree was constructed by using the Maximum-like methods with MEGA 6.0, and the tree was bootstrapped for 1000 replications.

### 4.6. Quantitative PCR (qPCR) 

The isolated total RNA was reverse-transcribed into single strand cDNA with Primescript™ First Strand cDNA Synthesis Kit (Takara, Kyoto, Japan). qPCR was performed on the LightCycle 480 II (Roche, Basel, Switzerland) using SYBR^®^ Premix Ex Taq™ II (Takara, Kyoto, Japan). The cycling conditions were: 94 °C for 5 min (min), followed by 40 cycles of 95 °C for 15 s (s), 58 °C for 15 s, 72 °C for 15 s [43]. The expression level was normalized by the 2^−ΔCT^ methods using *β-actin* as an internal control. The primers are listed in Table A1.

### 4.7. Plasmid Construction

The open reading frames (ORFs) of each *BsCXCR* were cloned into the pEGFP-N3 vector to generate GFP fusion proteins. The ORFs of *BsCXCR1a*/*b*, *BsCXCR3a1*/*2I,* or *BsCXCR7* were inserted into *EcoR* I/*BamH* I sites of the pEGFP-N3 vector; meanwhile, the ORFs of *BsCXCR4a*/*4b*, *BsCXCR5*, or *BsCXCR6* were inserted into *Nhe* I/*EcoR* I sites of the pEGFP-N3 vector. All plasmids were verified by DNA sequencing. The primers are listed in Table A1.

### 4.8. Immunofluorescence

HEK293T cells were seeded on a glass coverslip in 24-well culture plates. At 80% confluence, the cells were transfected with different *pEFP-N3-BsCXCR* plasmids using Lipofectamine 8000 (Beyotime, Shanghai, China). At 24 h post-transfection, cells were fixed in 4% paraformaldehyde for 20 min at room temperature (RT), washed in PBS three times, permeabilized in PBS with 0.2% Triton X-100 for 15 min, blocked with 5% milk, and incubated in anti-β-catenin antibodies with a dilution of 1:400 at 4 °C overnight. After three washes in PBST (0.1% Tween-20 diluted in PBS), and followed by three washes in PBS, the cells were incubated with Alexa Fluor^®^ 555 donkey-anti-mouse antibodies IgG H&L (Invitrogen, Carlsbad, CA, USA) at a dilution of 1:400 for 1 h at RT. Then, the cells were washed three times with PBS, and the nuclei were stained with DAPI (10 μg/L diluted in PBS solution) for 10 min. Finally, the cells were visualized and documented with confocal microscope (LSM800, Zeiss, Oberkohen, Germany).

### 4.9. Mechanical Skin Injury

A total of 30 four-eyed sleeper individuals weighing 15–20 g were used for mechanical skin injury experiments. After anesthetized with 1% MS222 (Sigma, Fullerton, CA, USA), mechanical skin injury wound (about 5 mm × 5 mm × 0.3 mm) was induced using surgical scissors on one side of posterior belly of fish, as previously described [35]. Then, the fish were cultured in sterile water. At 1 h, 3 h, 6 h, 12 h, and 24 h after injury, three fish per time point were anesthetized, and the skin tissue around the wounds were collected for RNA extraction. Each experiment has been repeated for three times.

### 4.10. Statistics Analysis

The statistics was calculated using SPSS version 20. Differences between control and treatment groups were assessed by one-way ANOVA. *p* < 0.05 was considered as statistically significance. 

## Figures and Tables

**Table 1:** Prediction of N-glycosylation sites in BsCXCR proteins.

Name	N-Glycosylation Sites	Count
*BsCXCR1a*	N^21^FTY, N^25^DSE (N-terminus), N^191^SSQ (ECL2)	3
*BsCXCR1b*	N^13^DTS, N^19^LTY (N-terminus), N^185^NSD, N^200^ASK (ECL2) N^351^GTV (C-terminus)	5
*BsCXCR2*	N^13^TSR, N^29^SSY, N^34^TSF (N-terminus), N^163^LSS (ICL2), N^209^LTG (ECL2), N^229^MTV (TM6), N^309^VTQ (ECL3), N^370^TSV (C-terminus)	8
*BsCXCR3a1*	N^23^DTW, N^31^YTV (N-terminus), N^90^WSV (ICL1), N^287^ITL (TM6), N^301^NTC (ECL3)	5
*BsCXCR3a2*	N^16^GSY (N-terminus), N^118^VSF (TM3), N^138^ITR (ICL2) N^262^TTL (TM6)	4
*BsCXCR4a*	N^14^DTG (N-terminus)	1
*BsCXCR4b*	N^13^SSE, N^17^LTD (N-terminus)	2
*BsCXCR5*	N^6^YTY, N^13^LTD (N-terminus), N^178^SSH (ECL2), N^273^ASC (ECL3), N^338^QSS (C-terminus)	5
*BsCXCR6*	N^11^YTQ, N^20^DST, N^29^SSD (N-terminus), N^366^EST (C-terminus)	4
*BsCXCR7*	N^21^ISE, N^28^IST (N-terminus), N^42^RSA, N^334^RTY, N^363^LSE (C-terminus)	5

## Data Availability

All the Illumina sequencing reads (SRA: PRJNA743360) and the sequence of 10 *BsCXCR* transcripts (Access no. MW239059-MW239068) have been deposited in the NCBI.

## Abbreviations

CXCR	CXC chemokine receptor
CCR	CC chemokine receptor
CX3CR	CX3C chemokine receptor
DAPI	4′,6-diamidino-2-phenylindole
DEGs	Differentially expressed genes
DMEM	Dulbecco’s modified eagle medium
ECL	Extracellular loop
GO	Gene ontology
ICL	Intracellular loop
KEGG	Kyoto encyclopedia of genes and genomes
KOG	Eukaryotic ortholog groups
ORF	Open Reading Frame
PBS	Phosphate buffered saline
qPCR	Quantitative polymerase chain reaction
RT	Room temperature
TM	Transmembrane domain
XCR	XC chemokine receptor
CXCL	CXC chemokine ligand

## Appendix A

**Table A1:** Sequences of primer used in this paper.

Name	Sequence (5′-3′)	Comments
*BsCXCR1a-F*	ACCGAATTCATGTCAGATGAAATCACCTCGTCTTTTAT	Plamid construction
*BsCXCR1a-R*	CGCGGATCCCATAAATGTCGATGTGATCTCTGAAGAC	
*BsCXCR1b-F*	ACCGAATTCATGTCTTTGTCTTTCGACTTCAGT	
*BsCXCR1b-R*	ACCGGATCCGAGAACTGTCCCATTGCTTTCTGAGG	
*BsCXCR3a1-F*	ACCGAATTCTCCTCAGTAATTCACCTGTTACT	
*BsCXCR3a1-R*	ACCGGATCCGATGGCAATGGAGTTGGAGGTGT	
*BsCXCR3a2-F*	ACCGAATTCTCCTCAGTAATTCACCTGTTACT	
*BsCXCR3a2-R*	ACCGGATCCGATGGCAATGGAGTTGGAGGTGT	
*BsCXCR4a-F*	ACCGCTAGCATGTCTTACTATGAGCACAT	
*BsCXCR4a-R*	CGCGGATCCACTGGAGTGTAGACTGGATGACTCG	
*BsCXCR4b-F*	ACCGCTAGCACTAACGTAACGTAACATCACGA	
*BsCXCR4b-R*	AGCGAATTCGAACTGGACAGCACACTTGAGGATTCCGA	
*BsCXCR5-F*	CTAGCTAGCATGGAGGAATCTGAGAATTATACATAT	
*BsCXCR5-R*	ACCGAATTCGAGAAGGTGTTGGTGGTTGTTGCTCC	
*BsCXCR6-F*	CTAGCTAGCATGACGTCTGTGTACC	
*BsCXCR6-R*	ACCGAATTCGACAAAATATTGCTGCCTGGAAACTTAAA	
*BsCXCR7-R*	ACCGAATTCGGTCAGTGGTCCTCTGTCAGC	
*BsCXCR7-R*	ACCGGATCCGATCTGCGGCGGATTGTCCAC	
*BsActin-F*	GACAGAGCGTGGCTACTCATTCACC	qRT-PCR
*BsActin-R*	GGTTTCATGGATACCGCAGGATTCC	
*BsCXCR1a RT-F*	AAGTGCTGAGGGGAGGAGTGAGGA	
*BsCXCR1a RT-R*	CATGAAATGGTGAAATGGAGAAATGGAG	
*BsCXCR1b RT-F*	CCTGACCATATGCCTCCTCC	
*BsCXCR1b RT-R*	ACAGAGCCATGAGACCATCC	
*BsCXCR3a1 RT-F*	CCTCTGAGGCTGTGAGTGGA	
*BsCXCR3a1 RT-R*	AGAACCACACCAGCAAGCAG	
*BsCXCR3a2 RT-F*	GGATGGTGCTGTGGTGTGTT	
*BsCXCR3a2 RT-R*	GCCTGGGTGTCTTCTGGGTA	
*BsCXCR4a RT-F*	CATAGTGTTGGGATGCCAGC	
*BsCXCR4a RT-R*	CAAGTAGCAGCTCCACAACG	
*BsCXCR4b RT-F*	CACGAGCGTTCAACATGGAT	
*BsCXCR4b RT-R*	CGATCTCTCACATGGCTCCT	
*BsCXCR5 RT-F*	GCTGCCTGAACCCTTTTCTC	
*BsCXCR5 RT-R*	AAGGTGTTGGTGGTTGTTGC	
*BsCXCR6 RT-F*	CTCCAAGCCACCCATACTCA	
*BsCXCR6 RT-R*	CACACACCGCAGCTATCTTC	
*BsCXCR7 RT-F*	GTACATTGCCCACTTAGCCG	
*BsCXCR7 RT-R*	GGAAGAAGATGCTGCCGAAG	
